# Rapid Profiling of Tumor‐Immune Interaction Using Acoustically Assembled Patient‐Derived Cell Clusters

**DOI:** 10.1002/advs.202201478

**Published:** 2022-05-25

**Authors:** Zheng Ao, Zhuhao Wu, Hongwei Cai, Liya Hu, Xiang Li, Connor Kaurich, Jackson Chang, Mingxia Gu, Liang Cheng, Xin Lu, Feng Guo

**Affiliations:** ^1^ Department of Intelligent Systems Engineering Indiana University Bloomington IN 47405 USA; ^2^ Division of Pulmonary Biology Center for Stem Cell and Organoid Medicine (CuSTOM) Cincinnati Children's Hospital Medical Center Cincinnati OH 45229 USA; ^3^ University of Cincinnati School of Medicine Cincinnati OH 45229 USA; ^4^ Department of Pathology and Laboratory Medicine Indiana University School of Medicine Indianapolis IN 46202 USA; ^5^ Melvin and Bren Simon Cancer Center Indiana University School of Medicine Indianapolis IN 46202 USA; ^6^ Department of Biological Sciences University of Notre Dame Notre Dame IN 46556 USA

**Keywords:** acoustofluidics, cell clusters, cancer immunity, patient‐derived tumor model, myeloid‐derived suppressor cells

## Abstract

Tumor microenvironment crosstalk, in particular interactions between cancer cells, T cells, and myeloid‐derived suppressor cells (MDSCs), mediates tumor initiation, progression, and response to treatment. However, current patient‐derived models such as tumor organoids and 2D cultures lack some essential niche cell types (e.g., MDSCs) and fail to model complex tumor‐immune interactions. Here, the authors present the novel acoustically assembled patient‐derived cell clusters (APCCs) that can preserve original tumor/immune cell compositions, model their interactions in 3D microenvironments, and test the treatment responses of primary tumors in a rapid, scalable, and user‐friendly manner. By incorporating a large array of 3D acoustic trappings within the extracellular matrix, hundreds of APCCs can be assembled within a petri dish within 2 min. Moreover, the APCCs can preserve sensitive and short‐lived (≈1 to 2‐day lifespan in vivo) tumor‐induced MDSCs and model their dynamic suppression of T cell tumor toxicity for up to 24 h. Finally, using the APCCs, the authors succesully model the combinational therapeutic effect of a multi‐kinase inhibitor targeting MDSCs (cabozantinib) and an anti‐PD‐1 immune checkpoint inhibitor (pembrolizumab). The novel APCCs may hold promising potential in predicting treatment response for personalized cancer adjuvant therapy as well as screening novel cancer immunotherapy and combinational therapy.

## Graphical abstract

Acoustically assembled patient‐derived cell clusters for the rapid profiling of complicated tumor microenvironment crosstalk are reported. This method is demonstrated to successfully model interactions among cancer cells, T cells, and short‐lived myeloid‐derived suppressor cells within 24 h. This method holds promising potential in predicting treatment response for personalized cancer adjuvant therapy and screening novel cancer treatments.

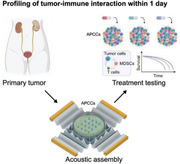

## 1 Introduction

Avoiding immune destruction is one of the emerging hallmarks of cancer.^[^ ^1^ ^]^ Immune suppressive cells such as myeloid‐derived suppressor cells (MDSCs) orchestrate local metabolism and cytokine environment to suppress anti‐tumor immunity and promote tumor growth.^[^ ^2^, ^3^, ^4^ ^]^ In turn, tumor cells actively reprogram the infiltrated immune cells and induce tumor microenvironment (TME) specific immune‐suppressive phenotypes,^[^ ^5^, ^6^, ^7^ ^]^ which are distinct from their circulating counterparts.^[^ ^8^, ^9^, ^10^, ^11^ ^]^ For example, when residing within the TME, MDSCs show distinct functions such as suppression of T cell‐mediated cytotoxicity via production of nitric oxide (NO) and arginase‐1 (ARG‐1).^[^ ^12^, ^13^, ^14^ ^]^ Clinically, higher numbers of MDSCs are generally associated with resistance to immune checkpoint inhibitor (ICI) treatments.^[^ ^15^, ^16^, ^17^ ^]^ Additionally, targeting MDSCs can overcome tumors’ resistance to ICI in animal studies.^[^ ^18^, ^19^, ^20^ ^]^

Currently, it remains a grand challenge to study human tumor‐induced MDSC functions and drug responses ex vivo due to their extremely short lifespan (≈1 to 2‐days). Once taken out of the context of the tumor microenvironment (TME), key phenotypes of tumor‐induced MDSCs such as NO and ARG‐1 production can be quickly dampened or lost.^[^ ^21^, ^22^ ^]^ Genetically engineered humanized mouse model may recapitulate human MDSC functions in vivo. However, they cannot fullyreflect the specific genetics and tumor heterogeneity of individual patients.^[^ ^23^, ^24^ ^]^ Despite partially preserving TME components, patient‐derived tumor organoids or organotypic cultures of tumor segments may lose lack key TME components such as the MDSCs, due to the lengthy culture time of 1–4 weeks.^[^ ^25^, ^26^, ^27^, ^28^ ^]^ Additionally, organotypic tumor cultures host a stochastic distribution of immune cells inside each tumor section/segment, thus hampering comparable evaluation of multiple drug treatments in parallel. Thus, there is a great need to develop patient‐derived tumor models that can preserve sensitive TME immune cell types (e.g., MDSCs) and enable massively parallel profiling of multiple drug treatments.

Here, we present novel acoustically assembled patient‐derived cell clusters (APCCs) for preserving original tumor and immune cell compositions, modeling their interactions in 3D microenvironments, and testing the treatment responses of patient primary tumors. By combining acoustic cell assembly and Matrigel immobilization, over 100 APCCs with uniformed cell composition and number can be generated and manipulated using primary patient tumor digests. We demonstrated that the APCCs preserved the viability and functional phenotypes of the MDSCs, including their expression of key immune suppressive genes,^[^ ^29^, ^30^ ^]^ inhibition of T cell‐mediated cytotoxicity,^[^ ^31^ ^]^ as well as inhibition of pro‐inflammatory cytokine secretion.^[^ ^21^ ^]^ Additionally, the APCCs were used to test the combinational treatment efficacy of MDSC‐targeting multi‐kinase inhibitor cabozantinib,^[^ ^32^, ^33^ ^]^ and anti‐PD1 drug pembrolizumab.^[^ ^34^ ^]^ By suppressing the MDSCs via cabozantinib treatment, we found the anti‐tumor effect of anti‐PD1 on renal cell carcinoma (RCC) patients’ primary tumors was enhanced by 123 ± 67%. Thus, we demonstrate this method could be widely applied to study tumor immunity ex vivo in a fast, scalable, and reproducible manner.

## 2 Results

### 2.1 Rapid Formation of Acoustically Assembled Patient‐Derived Cell Clusters

**Figure 1:**
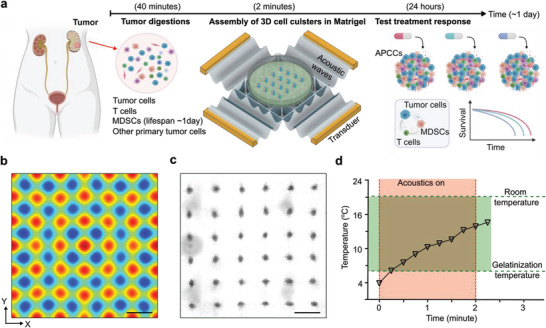
Acoustically assembled patient‐derived cell clusters (APCCs) for modeling tumor immunity and testing treatment within one day. a) The working flow of generating 3D APCCs within Matrigel using acoustic cell assembly for modeling MDSC/T cell/tumor cell crosstalk and testing treatment response. b) The simulation of acoustic field distribution for rapid assembly of tumor cells derived from a kidney cancer patient. c) The image of a large array of 3D cell clusters within Matrigel after acoustic assembly and Matrigel gelatinization. d) The temperature dynamics during a 2‐min acoustic cell assembly treatment and heat exchange within room temperature. Scale bar: 1 mm.

It is challenging to recapitulate the original tumor‐immune interaction within a primary tumor. Tumor cells and tumor‐associated immune cells could quickly change their phenotypes after dissociation.^[^ ^35^, ^36^ ^]^ One possible solution is to rapidly assemble all the cell components from primary tumor digests into 3D configurations, preserving the original tumor components, functional phenotypes, and tumor immune interactions (**Figure** **1**a). By coupling an acoustic cell assembly device with commercially available, sterile petri dishes, we could generate uniform acoustically assembled patient‐derived cell clusters (APCCs) within the extracellular matrix (Matrigel) in a fast, scalable, and highly biocompatible manner. The acoustic cell assembly device consisted of two pairs of piezoelectric transducers (PZTs) and a laser‐cut plastic holder. After being excited with the radio frequency signals, four sets of traveling acoustic waves can propagate and interfere with each other to form a large array of 3D acoustic traps.We simulated the acoustic distribution within the petri dish (Figure 1b). Single‐cell suspensions mixed with liquid Matrigel at 4 °C can be quickly aggregated into over 100 APCCs within a uniform array within a standard 30 mm cell culture petri‐dish after 2 min of acoustic assembly treatment (Figure 1c). After the 2 min acoustic treatment, cell clusters could be held in place by the gelatinized Matrigel. Matrigel is temperature‐sensitive, and the temperature increase by acoustic treatment and heat exchange at room temperature could convert the Matrigelfrom the liquid phase to the gel phase, holding all the assembled 3D cell clusters in place (Figure 1d). The diameters of the initial cell clusters range from 150 to 250 µm with corresponding input cell concentrations varying from 0.7 to 1.9 million mL^–1^ (Figure S1, Supporting Information). We chose to seed the cell suspensions at a concentration of 1.6 million cells per mL, which yields 2168 ± 206 cells per each APCC. Using the dissociated mouse syngeneic EO771 primary breast tumor as controls, we confirmed that the APCCs could preserve the original tumor microenvironment immune cells’ makeup, especially preserving the original T cells and MDSCs percentages (Figure S2, Supporting Information).

### 2.2 APCCs Preserve the Viability and Phenotypes of MDSCs

**Figure 2:**
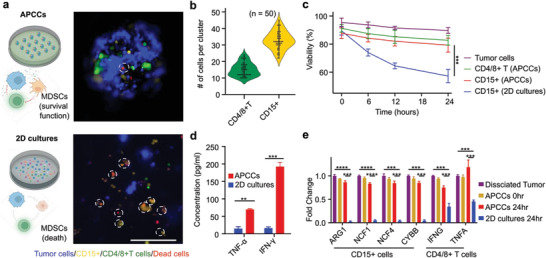
Acoustically assembled patient‐derived cell clusters (APCCs) versus 2D cultures. a) CD15^pos^ cell viability comparison between the APCCs and 2D culture conditions. CD15^pos^ cells were identified by DiL celltracker dye (yellow). CD15^pos^cell death were identified by overlapping of cell death indicator (red) and pre‐labeled CD15^pos^cells (dotted circle). b) Enumeration of CD15^pos^ cells and CD4/8^pos^ T cells within each APCC cluster. c) Quantification of tumor cells, CD15^pos^ cells, and CD4/8^pos^ T cells' viability within the APCCs and comparison with 2D culture conditions, *n* = 3. d) ELISA analysis of TNF‐*α* and IFN‐*γ* concentrations in culture supernatants from the 2D and APCCs cultures, *n* = 3. e) Gene expression analysis of CD15^pos^ cells and CD4/8^pos^ T cells in the 2D/APCCs conditions from dissociated tumors, at 0 h, and at 24 h post acoustic assembly, *n* = 3. Scale bar: 200 µm.

To evaluate the preservation of MDSC viability inside APCCs, we performed initial tests with RCC primary tumors from patients. We first isolated the CD15^pos^ cells from the dissociated patient tumor cells via magnetic microbeads, which consist of tumor‐infiltrating neutrophils including immunosuppressive polymorphonuclear myeloid‐derived suppressor cells (PMN‐MDSCs). We pre‐labeled the CD15^pos^ cells with DiL membrane dyes (shown as yellow). We also isolated T cells via a 1:1 mixture of CD4 and CD8 microbeads and pre‐labeled the T cells with DiO membrane dyes (shown as green). We also labeled the CD15^pos^, CD4^pos^, and CD8^pos^ depleted dissociated tumor cells with the blue CMAC cell tracker dye (shown as blue). Membrane impermeable cell nucleus staining NucRed dead 647 was added to indicate cell death (shown as red). We calculated the cell death of the CD15^pos^ cells by analyzing the co‐localization of DiL‐labeled MDSCs and NucRed cell death indicator dye over time‐lapse imaging (**Figure** **2**a). We analyzed the distribution of the CD15^pos^ cells and CD4/CD8^pos^ T cells across 50 APCCs. We found that CD15^pos^ cells and CD4/CD8^pos^ T cells showed relatively uniform distribution across these APCCs, with an average of 15 ± 4 CD4/CD8^pos^ T cells and 32 ± 5 CD15^pos^ cells within each APCC (Figure 2b). We further compared the viability of CD15^pos^ cells maintained in the 2D cultures and APCCs. We found that the CD15^pos^ cells maintained in the 2D dissociated tumor culture showed significant cell death over the 24 h in vitro culture time. The viability decreased significantly from 89.5 ± 3.4% at 0 h to 57.2 ± 4.7% at 24 h. In contrast, the CD15^pos^ cells inside APCCs showed minimal cell death upon acoustic treatment (viability before the 2 min acoustic treatment: 89.5 ± 3.4% versus viability after 2 min acoustic treatment: 87.8 ± 3.7%) and remained highly viable over 24 h culture period (24 h viability: 79.3 ± 5%) (Figure 2c). Furthermore, we analyzed cytokine secretion profiles from both the 2D cultures and APCCs. We found that the supernatants of APCCs showed a 12‐fold increase in IFN‐*γ* and a 4.8‐fold increase in TNF‐*α* concentrations as compared with 2D cultures (Figure 2d), demonstrating its capacity to better preserve the local cytokine microenvironment. Finally, we isolated the CD15+ cells and CD4/CD8^pos^ T cells at 24 h from APCCs and analyzed the expression of key immune‐suppressive genes of MDSCs (ARG1, NCF1, NCF4, CYBB) which mediate MDSC arginase 1 secretion and NO generation to inhibit T cells within the tumor TME. We found that while in the 2D cultures, ARG1, NCF1, NCF4, and CYBB expression were quickly lost in the CD15^pos^ cells, likely due to the lack of cell–cell contacts, these gene signatures were unaltered by the acoustic assembly and largely preserved over the 24 h culture period in the APCCs. Similarly, T cells' expressions of TNFA and IFNG were also better preserved by the APCCs as compared with 2D culture conditions (Figure 2e).

### 2.3 APCCs Model MDSC‐Mediated Tumor Immunosuppression

**Figure 3:**
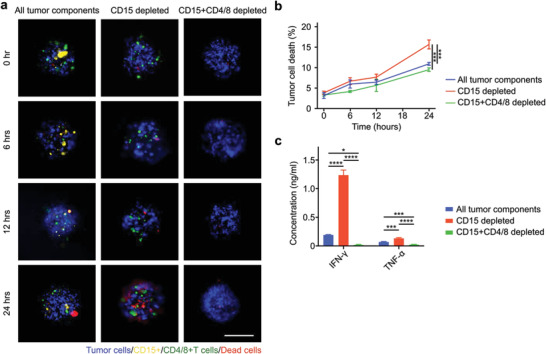
T cell cytotoxicity suppression by myeloid‐derived suppressor cells (MDSCs). a) Time‐lapse images of the acoustically assembled patient‐derived cell clusters (APCCs) with all tumor components (control), with CD15^pos^ cells removal and with CD15^pos^ cells plus CD4/8^pos^ T cells co‐removal conditions. b) Tumor cell death quantification within the APCCs upon control, CD15^pos^ cells removal, and CD15^pos^ cells plus CD4/8^pos^ T cells co‐removal conditions, *n* = 3. c) ELISA analysis of TNF‐*α* and IFN‐*γ* concentrations in culture supernatants from control, CD15^pos^ cells removal, and CD15^pos^ cells plus CD4/8^pos^ T cells co‐removal conditions, *n* = 3. Scale bar: 200 µm.

One of the main functions of MDSCs in tumors is that they inhibit T cell‐mediated tumor cell killing. To investigate whether this function is preserved in the APCCs, we set out to investigate the effect of MDSCs' removal on cell interactions within APCCs. To achieve this, we pre‐removed the CD15^pos^ cells (including PMN‐MDSCs) from dissociated tumor cells by magnetic‐based depletion. We observed tumor cell death by time‐lapse imaging within the control and CD15^pos^ cell‐removed APCCs. Tumor cell death was quantified by analyzing cells with colocalized signals of blue CMAC cell tracker dye labeling tumor cells and NucRed 647 far‐red dead cell labels (Figure S3, Supporting Information). We found that upon CD15^pos^ cell removal, tumor cell death within the APCCs increased by 43.4 ± 10.8% at 24 h (**Figure** **3**a,b). To further confirm that this enhanced tumor cell death was T cell‐dependent, we depleted the CD4/8^pos^ T cells together with the CD15^pos^ cells. Indeed, after the CD4/8^pos^ T and CD15^pos^ cells co‐removal, the tumor cell death was significantly reduced to a level even lower than the control conditions. We further investigated the cytokine microenvironment change upon the removal of CD15^pos^ cells. We found that the CD15^pos^ cells removal did enhance TNF‐*α* and IFN‐*γ* cytokine levels within the APCCs, as an indicator of potentially enhanced T cell activation. Furthermore, this increased cytokine secretion was also abolished upon T cell co‐removal (Figure 3c). Thus, we confirmed the portion of the CD15^pos^ cell inside APCCs preserved MDSC function to inhibit T cells.

### 2.4 APCCs Test MDSC‐Targeting and Combinational Treatments

**Figure 4:**
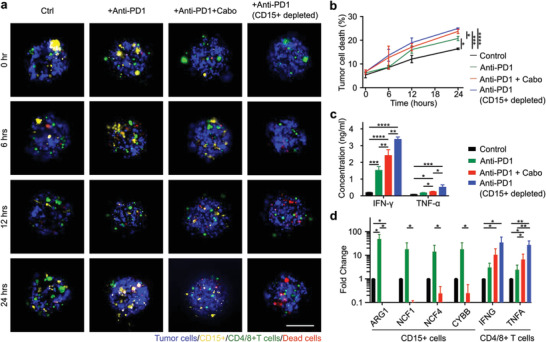
Evaluation of myeloid‐derived suppressor cells (MDSCs) targeting therapy. a) Time‐lapse images of the acoustically assembled patient‐derived cell clusters (APCCs) with control, anti‐PD1, anti‐PD1 combination with cabozantinib, and anti‐PD1 treatment with CD15^pos^ cells removal conditions. b) Tumor cell death quantification within the APCCs under control, anti‐PD1, anti‐PD1 combination with cabozantinib, and anti‐PD1 treatment with CD15^pos^ cells removal conditions, *n* = 3. c) ELISA analysis of TNF‐*α* and IFN‐*γ* concentrations in culture supernatants from control, anti‐PD1, anti‐PD1 combination with cabozantinib, and anti‐PD1 treatment with CD15^pos^ cells removal conditions, *n* = 3. d) qRT‐PCR analysis of purified MDSCs and T cells isolated from APCCs 24 h post‐treatment under control, anti‐PD1, anti‐PD1 combination with cabozantinib, and anti‐PD1 treatment with CD15^pos^ cells removal conditions, *n* = 3. Scale bar: 200 µm.

MDSC‐mediated immune evasion is a key mechanism underlying tumor development.^[^ ^37^, ^38^ ^]^ Thus, there is a growing interest to develop drugs targeting MDSCs to overcome ICI resistance. Therefore, we sought to validate our APCCs as a potential model to study the therapeutic effects of MDSC‐targeting drugs alone or in combination with ICIs. We dosed the APCCs derived from three RCC patients' primary tumors and observed the tumor cell death over a 24 h time‐lapse under the drug treatments of immune checkpoint inhibitor anti‐PD1 (pembrolizumab) alone or together with an MDSC‐targeting drug cabozantinib^[^ ^32^, ^33^ ^]^ (**Figure** **4**a). We observed that the anti‐PD1 treatment alone could enhance the tumor cell death by 17.0 ± 9.4% inside RCC‐derived APCCs as compared with untreated controls. When anti‐PD1 and cabozantinib treatments were combined, the tumor cell death within the APCCs was increased by 33.7 ± 6.5% as compared with the untreated control. This is a 123 ± 67% increase in drug‐induced tumor cell death as compared with anti‐PD1 treatment alone. Furthermore, we also interrogated the effect of anti‐PD1 treatments on APCCs with complete CD15^pos^ cells removal. This group yielded an even higher tumor cell death with an increase of 40.9 ± 9.4% as compared with the untreated control group (Figure 4b). Additionally, we also assayed the cytokine levels of TNF‐*α* and IFN‐*γ* within APCCs treated with anti‐PD1, anti‐PD1, and cabozantinib combination, as well as anti‐PD1 with CD15^pos^ cells' removal in RCC patient‐derived APCCs. We found that the levels of TNF‐*α* and IFN‐*γ* were both elevated in the anti‐PD1 treatment alone group, which was further elevated in the combinational treatment group as well as the MDSC removal group with anti‐PD1 addition. Finally, we analyzed MDSC suppressive gene expression inside APCCs upon drug treatments. We found that MDSCs suppressive genes were slightly upregulated in the anti‐PD1 treatment group, likely in response to enhanced T cell activity. But these suppressive gene expressions were almost completely abolished in the anti‐PD1 and cabozantinib combinational treatment group, suggesting cabozantinib effectively inhibited MDSCs' suppressive function. As for T cells, in concordance with ELISA data, TNFA and IFNG gene expressions were enhanced by anti‐PD1 treatment, which was further augmented by combinational therapy with cabozantinib, and were highest in the CD15^pos^ cells removal + anti‐PD1 treatment group.

## 3 Discussion

Solid tumors and other solid tissues often host a tissue‐specific milieu that reprograms infiltrating immune cells to induce a tissue‐specific phenotype, such as mucosal‐associated invariant T cells (MAITs),^[^ ^39^ ^]^ brain resident meningeal macrophages (MGMs),^[^ ^40^ ^]^ and tumor‐induced MDSCs.^[^ ^41^ ^]^ Once taken out of the milieu, the in situ phenotypes of these tissue‐resident immune cells are often quickly lost due to a diluted paracrine cytokine environment and lack of cell‐cell interactions. In this work, we reported acoustically assembled patient‐derived cell clusters (APCCs) to quickly reconstitute the local cell–cell interactions and paracrine factors in a fast, high‐throughput, and label‐free manner. By applying bio‐compatible bulk acoustic waves onto dissociated tumor cell suspensions, we were able to quickly aggregate cells into compact, uniform clusters, thus preserving the original tumor compositions.

We demonstrated the advantage of our APCCs by showing the preservation of viability and functional phenotypes of a microenvironment sensitive, difficult to culture TME niche cell type: tumor‐induced MDSCs. MDSC is a key immune suppressive cell type within the TME that mediates resistance to ICI treatments.^[^ ^42^ ^]^ There has been growing interest to target MDSCs in ICI‐resistant or refractory solid tumors.^[^ ^43^ ^]^ Additionally, MDSCs have also been shown to play key roles in non‐tumor settings such as acute bacterial and viral infection,^[^ ^44^, ^45^ ^]^ as well as autoimmune diseases.^[^ ^46^ ^]^ However, traditional 2D cultures of isolated MDSCs will lead to rapid cell death as well as loss of key phenotypes. Our results showed that benefiting from the fast (2 min), label‐free acoustic assembly, our APCCs could not only preserve the viability of MDSCs but also their phenotypes including expression of arginase and NO production‐related genes, which were otherwise quickly lost in 2D culture. We demonstrated that the MDSCs inside these APCCs could also function to inhibit T cell mediated‐tumor cell death in vitro. Additionally, we demonstrated that the APCCs could serve as a model to test existing drugs’ effects on MDSC functions that can be potentially applied for cancer immunotherapy. Many previously FDA‐ approved drugs were initially intended for other applications/indications. For example, cabozantinib tested in our study was initially approved to treat advanced renal carcinoma for its anti‐angiogenesis activity.^[^ ^47^ ^]^ Our data demonstrated in human system that cabozantinib could also function through inhibition of PMN‐MDSC activity, echoing previous reports in animal models.^[^ ^18^, ^48^ ^]^ This indicated the potential of our model to screen for other FDA‐approved drugs to elucidate their TME modulation functions for novel drug applications and discoveries. Together, our results indicated that the APCCs could preserve cellular and functional profiles of sensitive immune cell populations such MDSCs within the TME.

Patient‐derived tissues/cells hold important genetic, epigenetic, transcriptomic, and proteomic information unique to the patient's genetics and the treatments they received. Profiling of patient‐derived tissue has shown promising potential for disease diagnosis, prognosis, and precision medicine. Traditional molecular profiling technologies, such as DNA/RNA sequencing, treat tumors as bulk tissue and lack information regarding functional phenotypes of specific cell types within the tissues. Single‐cell profiling technologies such as CyTOF or single‐cell sequencing could provide information regarding subpopulations of tissue cells. However, they are rather expensive and cannot be used to study drug treatment responses directly. Patient‐derived xenograft (PDX) models can be used for individualized drug treatment studies. However, PDX requires large quantities of tissues and is not amenable to high throughput screening studies, and cannot be used to study immune components within the original tissue. Our APCCs provide an alternative solution where we could preserve original tissue make‐up in acoustic assembled 3D cultures with rapid profiling of drug treatment responses with data readout within 24 h. This not only enables MDSC studies to explore their biological functions ex vivo utilizing patient‐derived cells but also shows promise for applications that require preservation of localized, complex cell–cell communications, such as modeling lymph node immunity^[^ ^49^ ^]^ and inflammatory hot‐spots known as tertiary lymphoid structures (TLS) in various organs.^[^ ^50^ ^]^ Overall, our method provides a novel tool for studying localized cell–cell interactions in a rapid manner which preserved the cell phenotypes and their responses to treatment. One limitation of our current APCCs would be that they lack representation of other aspects of TME such as tumor vasculature, crosstalk with peripheral immune systems as well as dynamic evolution in response to treatments. To improve our APCCs, we could further develop our models to incorporate tumor vasculature chips with immune cell perfusion in the future to better capture these aspects of tumor biology. Additionally, tumor metastasis could constitute distinct TME as compared with the primary tumor, future efforts could incorporate co‐culture experiments using APCCs with tissue type‐specific organoids (e.g., lung, brain, and liver organoids) to study tumor cells behavior and crosstalk with other organs.

To conclude, our novel APCCs is a label‐free, cell‐type agnostic tool made to study localized cell–cell paracrine signaling and interactions in a standardized, uniform, and scalable manner. Our APCCs have the broad potential for immunotherapy screening applications as well as the specialized utility as companion diagnostics for precision medicine.

## 4 Experimental Section

### Device Fabrication

This acoustic platform consisted of an inner PMMA holder chamber of 40 mm × 40 mm to hold a 30 mm diameter sterile cell culture petri‐dish and four flanking slots of 20 mm × 5 mm to hold two pairs of piezoelectric transducers.

### Digestion of Patient Primary Tumors

Fresh patient RCC tumors were collected by the tissue procurement and distribution core, at Indiana University Melvin and Bren Simon Comprehensive Cancer Center. The study is approved under IRB protocol # 11 280. The Tissues were weighed and digested using the gentleMACS dissociator (Miltenyi) following instructions of the Miltenyi human tumor dissociation kit.

### Acoustic Assembly of Patient‐Derived Tumor Cells

For acoustic cell assembly, a sterile 30 mm diameter petri‐dish holding 200 µL of cell suspensions at 1.6 million cells per mL was inserted into the inner PMMA chamber. Sterile deionized water was then carefully added to the space between the petri‐dish and PMMA chamber to conduct acoustics. To assemble cell suspensions into APCCs, 200 µL of patient primary tumor‐derived single‐cell suspensions in Matrigel (Corning) (37.5% v/v in RPMI‐1640 culture medium, Gibco) was added to the sterile petri dish. Signal inputs (0.996 and 1.006 MHz) with 20% duty cycle and 200 mVpp were applied to two pair transducers, respectively. After 2 min, cells were aggregated into clusters with uniform sizes by the acoustic radiation force. Meanwhile, the temperature was raised to 24 °C, leading to gelation of the Matrigel, fixing the cell clusters in place. The acoustics were then turned off and the petri‐dish was left on stage for 30 min to allow the Matrigel to fully gelatinize. The cells in the petri‐dish were then topped with 100 µL of pre‐warmed complete medium (RPMI‐1640 + 10% fetal bovine serum (FBS) + 1X MEM Non‐Essential Amino Acids supplement + 1X GlutaMAX, Gibco) and transferred into a 37 °C on‐stage incubation chamber (Tokai‐hit) or standard cell culture incubator for continuous culture. Similarly, for 2D controls, the same volume of cells (200 µL) at the same concentration (1.6 million cells per mL) was added to a 30 mm diameter petri‐dish and topped with an additional 100 µL of pre‐warmed culture medium. The 2D controls were set up at the same time as APCCs and transferred to the incubator at the same time.

### Isolation and Labeling of Tumor Immune Components

Digested tumor cells were equally divided into three tubes. First tube: the CD15^pos^ cells were labeled with CD15 magnetic beads (Miltenyi) and were isolated by positive selection. Second tube: T cells were labeled with CD4 and CD8 magnetic beads (1:1) (Miltenyi), and were isolated by positive selection. Third tube: All three markers were labeled with magnetic beads (CD15, CD4, CD8) and both CD15^pos^ cells and CD4/8+ T cells depleted by negative selection to receive CD15^pos^ and CD4/8+ T cells co‐depleted tumor components. The T cells and CD15^pos^ cells were resuspended in RPMI‐1640 medium (serum‐free) at 1 million cells per 1 mL to stain with Vybrant DiO and DiL (Invitrogen) at 1:1000 dilutions at 37 °C for 1 h. T cells and CD15^pos^ cells co‐depleted tumor components were resuspended in complete RPMI‐1640 medium at 1 million cells per 1 mL and stained with CellTracker Blue CMAC dye (Invitrogen) at 1 µm at 37 °C for 1 h. Labeled CD4/8+ T cells, CD15^pos^ cells, and CD4/8+ CD15 co‐depleted tumor components were then washed twice in a complete RPMI‐1640 medium and mixed back. The cell mixtures were then resuspended at 1.6 million cells per mL in 37.5% Matrigel in pre‐chilled complete RPMI‐1640 medium v/v to prepare for acoustic assembly. Before acoustic assembly, one drop of NucRed Dead 647 Ready Probes (Invitrogen) was added to 1 mL of cell suspensions and left in a medium to visualize cell death overtime.

### Flow Cytometry Analysis of the Mouse Primary Tumors and APCCs

Orthotopic mouse tumors were digested using a mouse tumor dissociation kit (Miltenyi). Digested single‐cell suspensions were resuspended in a flow buffer made with filtered 1X phosphate‐buffered saline (PBS, Gibco), supplemented with 10% FBS (Gibco). Cell suspensions were then assembled into APCCs. For flow cytometry labeling, cells were then retrieved from APCCs by gentle digestion with cell recovery solution (Corning) and resuspended in flow buffer. The cells were then labeled with the corresponding fluorophore‐conjugated antibodies (Table S1, Supporting Information) for 30 min at 4 degrees. The labeled cells were washed twice with a flow buffer and analyzed using a BD LSRII flow cytometer. Compensation controls were prepared using anti‐rat or anti‐mouse compensation particles (BD) and run together with the samples.

### Drug Treatment of the APCCs

For drug treatments, anti‐PD1 antibodies (pembrolizumab, SelleckChem) were added at 5 µg mL^–1^ to the APCCs. Cabozantinib (SelleckChem) was added at 10 µm to the APCCs.

### qRT‐PCR Analysis of the MDSCs and T cells

For qRT‐PCR analysis of the CD15^pos^ cells and CD4/8^pos^ T cells, CD15^pos^ cells or CD4/8^pos^ T cells were pre‐labeled by CD15 or CD4 and CD8 (1:1) magnetic beads for 15 min at 4 °C, respectively, before being mixed with other tumor cells for acoustic assembly. The CD15^pos^ cells or CD4/8+ T cells with the labeling beads were then assembled into APCCs. To analyze their gene expression profiles, the APCCs were digested with cell recovery solution (Corning), and input onto MACS columns (Miltenyi) for positive magnetic separation to isolate pure CD15^pos^ cells or T cells (CD4^pos^ or CD8^pos^). The purified CD15^pos^ cells or CD4/8 + T cells were then lysed for RNA extraction by RNeasy Mini Kit (Qiagen), reverse transcribed to cDNA by High‐Capacity cDNA Reverse Transcription Kit (Applied Biosystems), and analyzed using corresponding primer pairs (Table S2, Supporting Information) and SYBR Green PCR Master Mix (Applied Biosystems). The samples were run on a StepOne Real‐Time PCR machine (Applied Biosystems) to quantify signals. The results were analyzed using the ∆∆Ct method for fold change analysis.

### ELISA Analysis of Cytokines

To perform cytokine analysis on the APCCs and 2D tumor cultures, 50 µL supernatants were pipetted out of the culture vessel and centrifuged at 10 000 *g* for 10 min to remove cell debris. The supernatants were then analyzed by human TNF‐*α* or IFN‐*γ* kits (Tribioscience).

### Statistical Analysis

All data were extracted and analyzed using Prism 7 (GraphPad Software). The *p*‐value between two samples was analyzed by student's *t*‐tests. *p*‐Value among three or more samples was analyzed by one‐way ANOVA followed by Tukey's honestly significant difference (HSD) post hoc test. *p*‐Values were denoted as following: * < 0.05; ** < 0.01; *** < 0.005; **** < 0.001.

## Conflict of Interest

The authors declare no conflict of interest.

## Supporting information

Supporting Information

## Data Availability

The data that support the findings of this study are available from the corresponding author upon reasonable request.
