# Supplementary material for: Rapid Profiling of Tumor‐Immune Interaction Using Acoustically Assembled Patient‐Derived Cell Clusters
Source: Adv Sci (Weinh). 2022 May 25;9(22):2201478. doi: 10.1002/advs.202201478 (PMC9353481; doi:10.1002/advs.202201478)
Supplement: Supplementary file 1 — Supporting Information [file ADVS-9-2201478-s001.pdf]

ADVANCED SCIENCE
Open Access

Supporting Information

for *Adv. Sci.*, DOI 10.1002/advs.202201478

Rapid Profiling of Tumor-Immune Interaction Using Acoustically Assembled Patient-Derived Cell Clusters

*Zheng Ao, Zhuhao Wu, Hongwei Cai, Liya Hu, Xiang Li, Connor Kaurich, Jackson Chang, Mingxia Gu, Liang Cheng, Xin Lu and Feng Guo**

Supplementary Information

Rapid profiling of tumor-immune interaction using acoustically assembled patient-derived cell clusters

Zheng Ao,[1] *Zhuhao Wu,*[1] *Hongwei Cai,*[1] *Liya Hu,*[1] *Xiang Li,*[1] *Connor Kaurich,*[1] *Jackson Chang,*[1] *Mingxia Gu,*[2,3] *Cheng Liang,*[4] *Xin Lu,*[5,6] *and Feng Guo,*[1,5*]

1. Department of Intelligent Systems Engineering, Indiana University, Bloomington, IN 47405, United States
2. Perinatal Institute, Division of Pulmonary Biology, Cincinnati Children's Hospital Medical Center, Cincinnati, OH 45229, United States
3. Center for Stem Cell and Organoid Medicine, CuSTOM, Division of Developmental Biology, Cincinnati Children's Hospital Medical Center, Cincinnati, OH 45229, United States
4. Department of Pathology and Laboratory Medicine, Indiana University School of Medicine, Indianapolis, IN 46202, United States
5. Melvin and Bren Simon Cancer Center, Indiana University School of Medicine, Indianapolis, IN 46202, United States
6. Department of Biological Sciences, University of Notre Dame, Notre Dame, IN 46556, United States

*Corresponding email: fengguo@iu.edu

Supplementary Figures

- **Figure S1** Optimization of the acoustic field for patient-derived cells assembly
- **Figure S2** Immune cell profiling of tumor microenvironment (TME) components of the dissociated primary tumor and acoustically assembled patient-derived cell clusters (APCCs)
- **Figure S3** Quantification of MDSCs viability in APCCs under control and treatment conditions

Supplementary Tables

- **Table S1** Antibody used in flow cytometry analysis of mouse EO771 primary tumors
- **Table S2** Primer sequences for qPCR analysis
- **Table S3** Patient demographics

Supplementary Figures

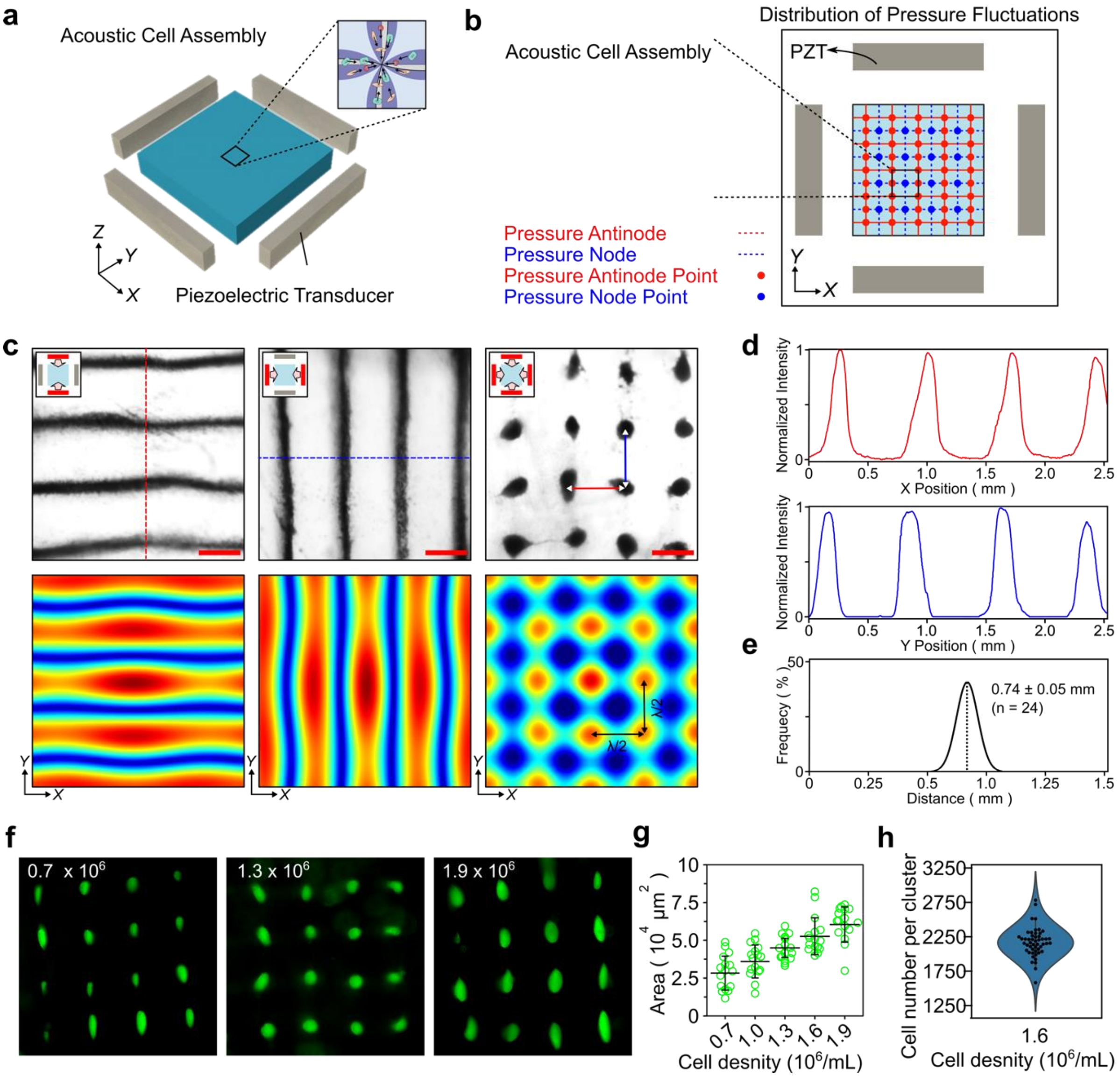

Figure S1 Optimization of the acoustic field for patient-derived cells assembly. (a) Schematics showing a configuration of acoustic devices. **(b)** Schematics of acoustic field inside cell culture chamber. **(c)** Simulation and results of acoustic fields assembling polystyrene (PS) beads with single pair of piezoelectric transducers (PZT) or both pairs of PZT turned on. **(d)** Distribution of PS beads along the X and Y axis of acoustic fields. **(e)** Distribution of distances between neighboring acoustic pressure antinodes. **(f)** Varying sizes of cell clusters can be formed by acoustic assembly input cell concentrations were changed. **(g)** Quantification of cell cluster sizes with corresponding input cell concentrations. **(h)** Quantification of cell number per cluster in the 1.6X10^6 cells/mL seeding conditions. Scale bar: 1 mm.

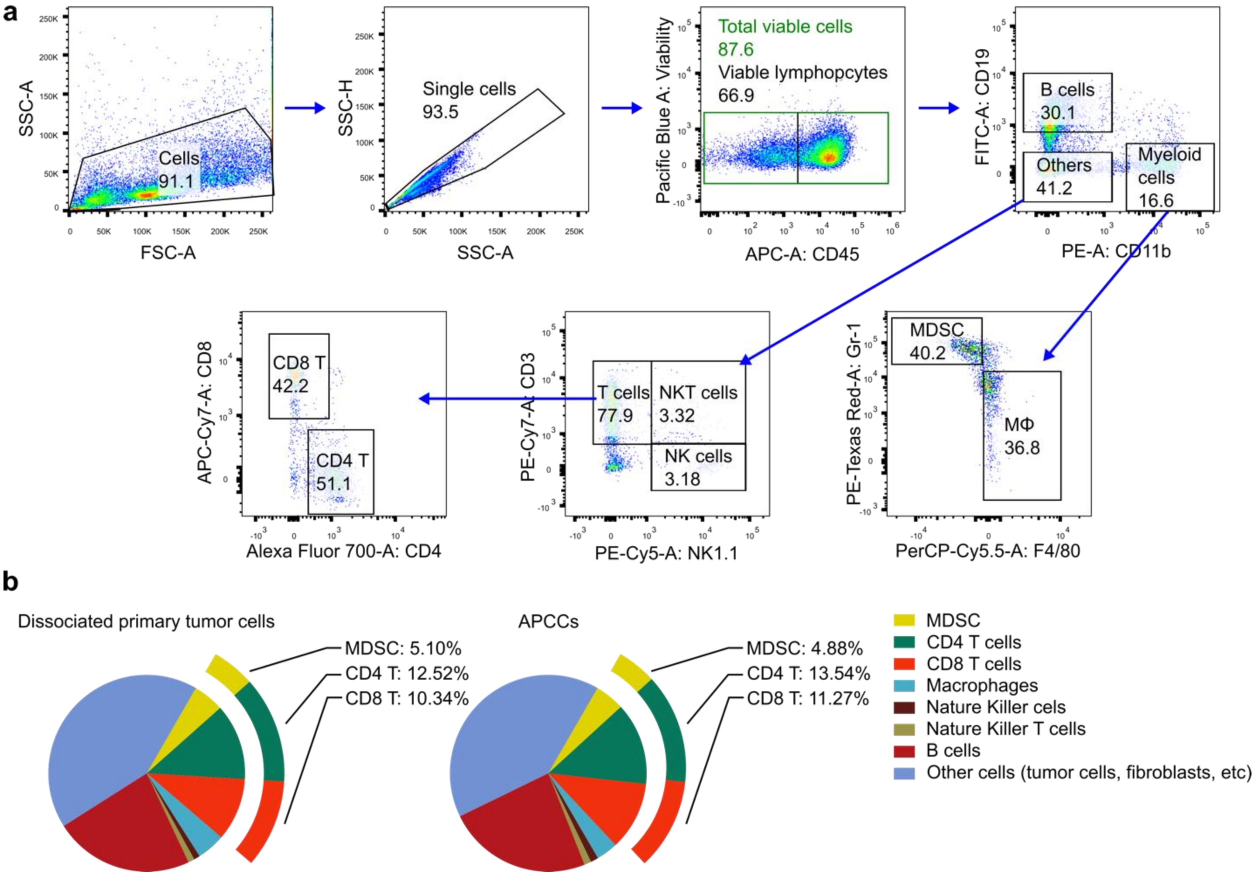

Figure S2 Immune cell profiling of tumor microenvironment (TME) components of dissociated EO771 mouse primary tumor and acoustically assembled cell clusters (APCCs). (a) Gating strategy to analyze TME immune cell components. **(b)** Comparison of T cell and myeloid-derived suppressor cells (MDSCs) makeup in dissociated primary tumor cells and APCCs. Cell components percentage are calculated as corresponding cell count/ total viable cell count. All animal experiments and procedure are approved by Indiana University Bloomington Institutional Animal Care and Use Committee (BIAUC) with an approval number of # 16-022-20.

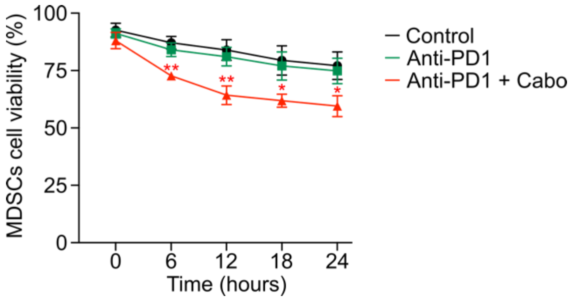

Figure S3 Quantification of MDSCs viability in APCCs under control and treatment conditions. Viability of MDSCs were quantified by analyzing colocalized MDSC labeling dye DiL (red) and cell death indicator NucRed Dead 647 (far-red) under control, anti-PD1 single agent and anti-PD1+Cabozantinib co-treatment conditions. Statistical analysis was performed by one-way ANOVA followed by post-hoc Tukey's tests. *: $p<0.05$; **: $p<0.01$.

Supplementary Tables

Table S1 Antibody used in flow cytometry analysis of mouse EO771 primary tumors

Antigen	Fluorophore	Host	Vendor	Catalog#	Dilution
CD45	APC	Rat	Biolegend	103111	1:100
CD3	PE/Cy7	Rat	Biolegend	100219	1:200
CD4	Alexa Fluor 700	Rat	Biolegend	100429	1:200
CD8a	APC/Cy7	Rat	Biolegend	100713	1:200
CD19	FITC	Rat	Biolegend	152403	1:100
CD11b	PE	Rat	Biolegend	101207	1:200
F4/80	PerCP/Cy5.5	Rat	Biolegend	123128	1:200
NK1.1	PE/Cy5	Mouse	Biolegend	108715	1:100
Gr-1	PE-eFluor 610	Rat	Invitrogen	61-5931-82	1:100

Table S2 Primer sequences for qPCR analysis

Gene	Primer sequence
hGAPDH_Fwd	AGGTCGGAGTCAACGGATTT
hGAPDH_Rev	TTCCCGTTCTCAGCCTTGAC
hArg1_Fwd	ACGGAAGAATCAGCCTGGTG
hArg1_Rev	GGCACATCGGGAATCTTTCCT
hNCF1_Fwd	GAGTACCGCGACAGACATCA
hNCF1_Rev	CGCTCTCGCTCTTCTCTACG
hNCF4_Fwd	AGAAGAGAGGCTTCACCAGCCA
hNCF4_Rev	TCCTCCAGCTTGCTCTGCAAAG
hCYBB_Fwd	CTCTGAACTTGGAGACAGGCAAA
hCYBB_Rev	CACAGCGTGATGACAACTCCAG
hTNFA_Fwd	CTCTTCTGCCTGCTGCACTTTG
hTNFA_Rev	ATGGGCTACAGGCTTGTCACTC
hIFNG_Fwd	GAGTGTGGAGACCATCAAGGAAG
hIFNG_Rev	TGCTTTGCGTTGGACATTCAAGTC

Table S3 Patient demographics

#	Diagnosis	Age	Race	Sex	Surgery
Patient 001	Renal cell carcinoma	74	Caucasian	Male	Right nephrectomy
Patient 002	Renal cell carcinoma	64	Caucasian	Male	Left nephrectomy
Patient 003	Renal cell carcinoma	52	Caucasian	Male	Right nephrectomy
